# The *Babesia bovis hap2* gene is not required for blood stage replication, but expressed upon *in vitro* sexual stage induction

**DOI:** 10.1371/journal.pntd.0005965

**Published:** 2017-10-06

**Authors:** Hala E. Hussein, Reginaldo G. Bastos, David A. Schneider, Wendell C. Johnson, Fatma K. Adham, William C. Davis, Jacob M. Laughery, David R. Herndon, Heba F. Alzan, Massaro W. Ueti, Carlos E. Suarez

**Affiliations:** 1 Department of Veterinary Microbiology and Pathology, Washington State University, Pullman, WA, United States of America; 2 Department of Entomology, Faculty of Science, Cairo University, Giza, Egypt; 3 Animal Disease Research Unit, USDA-ARS, Pullman, WA, United States of America; 4 Parasitology and Animal Diseases Department, National Research Center, Egypt; Yale University, UNITED STATES

## Abstract

*Babesia bovis*, is a tick borne apicomplexan parasite responsible for important cattle losses globally. *Babesia* parasites have a complex life cycle including asexual replication in the mammalian host and sexual reproduction in the tick vector. Novel control strategies aimed at limiting transmission of the parasite are needed, but transmission blocking vaccine candidates remain undefined. Expression of HAP2 has been recognized as critical for the fertilization of parasites in the *Babesia*-related *Plasmodium*, and is a leading candidate for a transmission blocking vaccine against malaria. Hereby we identified the *B*. *bovis hap2* gene and demonstrated that it is widely conserved and differentially transcribed during development within the tick midgut, but not by blood stage parasites. The *hap2* gene was disrupted by transfecting *B*. *bovis* with a plasmid containing the flanking regions of the *hap2* gene and the *GPF-BSD* gene under the control of the *ef-1α-B* promoter. Comparison of *in vitro* growth between a *hap2-*KO *B*. *bovis* clonal line and its parental wild type strain showed that HAP2 is not required for the development of *B*. *bovis* in erythrocytes. However, xanthurenic acid-*in vitro* induction experiments of sexual stages of parasites recovered after tick transmission resulted in surface expression of HAP2 exclusively in sexual stage induced parasites. In addition, *hap2*-KO parasites were not able to develop such sexual stages as defined both by morphology and by expression of the *B*. *bovis* sexual marker genes 6-Cys *A* and *B*. Together, the data strongly suggests that tick midgut stage differential expression of *hap2* is associated with the development of *B*. *bovis* sexual forms. Overall these studies are consistent with a role of HAP2 in tick stages of the parasite and suggest that HAP2 is a potential candidate for a transmission blocking vaccine against bovine babesiosis.

## Author summary

*Babesia bovis*, is a tick borne apicomplexan parasite responsible for important cattle losses globally. *Babesia* parasites have a complex life cycle including asexual replication in the mammalian host and sexual reproduction in the tick vector. Novel control strategies aimed at limiting transmission of the parasite are needed, but transmission blocking vaccine candidates remain undefined. In this study we analyze the conservation and role of the *hap2* gene in the erythrocyte stage of the life cycle of the parasite and found that expression of the gene is not required for the development of the parasite in erythrocytic stages, using a *hap2* mutated parasite line. In addition, we developed an *in vitro* system for the induction of sexual forms of *B*. *bovis* and found expression of the *hap2* gene and surface localization of the protein. However, *hap2*-KO parasites are unable to develop sexual stages. We concluded that HAP2 is a leading candidate for a transmission blocking vaccine against bovine babesiosis due of the high level of conservation, surface exposure, and specific expression in tick stage and in *in vitro* induced sexual stages parasites.

## Introduction

Bovine babesiosis is a tick-borne disease that limits food production in tropical and subtropical regions worldwide. The disease is mainly caused by *Babesia bovis*, *B*. *bigemina*, and *B*. *divergens* and is endemic in large parts of Australia, Africa, Asia, Europe, and Latin America [1]. Parasites of the genera *Babesia* are transmitted by ixodid ticks including *Rhipicephalus spp* [2–4]. Animals that survive acute infection remain persistently infected and are reservoirs for tick transmission [5, 6]. Bovine babesiosis control strategies have been met with limited success in some countries. However, these strategies, including acaricide treatment and live, attenuated vaccines [1, 7–9], are restricted due to increasing acaricide-resistant tick populations and by practical constraints of the live *Babesia* vaccines, such as possible reversion to virulence and the risk of tick transmission [7, 10, 11]. Despite safety concerns, some countries in endemic regions still use live vaccines to mitigate acute infection and prevent mortality.

To complete its life cycle, *Babesia* may require strict regulation of gene expression to develop, invade, replicate and survive in distinct and diverse hosts and tick vectors. *Babesia* parasites have a complex life cycle including asexual replication of haploid stages in the mammalian hosts and sexual reproduction of diploid stages in the tick vector [12]. The initial phenotypic differentiation of *Babesia* into sexual stages that occurs in the tick midgut lumen may require the expression of a subset of proteins necessary for fusion and formation of diploid zygotes [13]. Zygotes selectively infect tick midgut epithelial cells and subsequently develop into kinetes [14]. Mature kinetes are released into the tick hemocoel and invade various tick organs including salivary glands and ovaries. Eventually, the parasite is vertically transmitted to the next tick generation where another morphological change occurs as the parasite transforms into sporozoites in larval salivary gland acinar cells [12].

In a closely related human pathogen, *Plasmodium* specific proteins have been identified with important functions for parasite development within mosquitos. *Plasmodium* expresses a protein known as HAPLESS2/GCS1 (HAP2) and it is exclusively expressed on the surface of microgametes that occur in the mosquito gut lumen [15]. This protein is critical for the fertilization of parasites prior to development of the stage that infects mosquito gut epithelial cells. In *Plasmodium*, HAP2 is a candidate for a transmission blocking vaccine. Similar to *Plasmodium*, fertilization of *B*. *bovis* gametes within the vector tick midgut lumen is an obligate step for the parasite to perpetuate its life cycle. Disruption of *B*. *bovis* fertilization during parasite development in tick midgut would prevent transmission via tick vectors. Recent research described additional members of the *B*. *bovis* 6-Cys genes and defined the 6-Cys *A* and *B* genes as markers for midgut stages [16]. However, little else is known regarding the expression of additional sexual stage proteins by *B*. *bovis*, or the events leading to sexual reproduction of the parasite during its development in the midgut. *In silico* analysis demonstrated the presence of a gene in *B*. *bovis* genome that is orthologous to the *Plasmodium hap2* gene. The pattern of expression, localization and biological significance of HAP2 in *B*. *bovis* remains unknown. In this study, we demonstrate that *hap2* is transcribed exclusively during *B*. *bovis* development within the tick midgut, and not by blood stage parasites. We also demonstrated that deletion of *hap*2 does not affect the growth of *B*. *bovis* blood stages in cultures, and that the expression of HAP2 is associated to sexual stage development in *in vitro* sexual stage induction experiments. Collectively, the data indicates that similar to *Plasmodium* [17], *B*. *bovis* HAP2 is a potential candidate antigen for developing transmission blocking vaccines that might elicit a host immune response able to disrupt the development of a *B*. *bovis* stage infectious for tick midgut epithelial cells.

## Materials and methods

### Pattern of *hap2* gene expression by *B*. *bovis* blood and tick stages

To examine the pattern of *hap2* expression in *B*. *bovis* infected tick midgut, approximately 20,000 *Rhipicephalus microplus* larvae, La Minita strain, were placed under a cloth patch on a splenectomized calf as previously described [18, 19]. When approximately 1% of the nymphs molted to adults, the calf was inoculated intravenously with *B*. *bovis* Texas strain stabilate contained 10^7^ infected erythrocytes [20] to synchronize peak parasitemia with female tick repletion. Replete female ticks were collected, washed in tap water, dried and incubated at 26°C in 93% relative humidity. During development of *B*. *bovis* within tick midgut, five engorged ticks from the incubator were dissected daily for 6 consecutive days. Individual midgut was placed into 1 ml of Trizol (Thermo Fisher Scientific, Waltham, MA) and stored at -80°C. To evaluate *hap2* expression in *B*. *bovis* blood stages, infected defibrinated blood was collected and the erythrocytes washed five times with Puck’s saline G to remove white blood cells. Parasites were pelleted by centrifugation of infected blood and suspended in Trizol. To extract RNA from *in vitro* sexual stages induced culture, parasites were isolated by differential centrifugation of 400 *xg* to 2,000 *xg* to pellet the extracellular stages. Total RNA extracted using the Trizol according to the manufacturer’s protocol. The samples were treated with DNase I, quantified by Nanodrop (Thermo Fisher Scientific), and 150 ng of total RNA utilized for synthesizing cDNA (Thermo Fisher Scientific). Primer sets for *hap2* were designed to amplify a 165 base pair fragment (Table 1). PCR cycling conditions consisted of 95°C for 3 min followed by 35 cycles of 95°C for 30 sec, 55°C for 30 sec and 72°C for 30 sec, with a final extension of 72°C for 5 min. PCR products were visualized by 2% agarose gel electrophoresis. The PCR amplicon was cloned into PCR 2.1-TOPO (Thermo Fisher Scientific) and submitted for sequencing (Eurofins MWG Operon, Louisville, KY).

**Table 1:** Primers used to amplify the target genes.

Target	Primer forward (5’-3’)	Primer reverse (5’-3’)	Product size (bp)
*hap2 full length*	atggatggacccgagaagcgt	caatgggattggatcccgacg	2271
*hap2-5’flanking region*	ggggctcgagctagaaacccctcaa	ccccctcgagtacatcaacgttggtg	938
*hap2-3’flanking region*	gggggatcctgtatagagagcacaa	cccggatccatactcttaaactact	627
*hap2/ RT- PCR*	aaagcgtctatgtaatcaa	acagttttcttctcgtca	165
*6-Cys A/ RT- PCR*	atggatatccaaaacacattaaatagg	caacatattattcctgtccacacc	558
*6-Cys B/ RT- PCR*	atgtcgcaattaaacttac	atgtggagtatccgggcc	500

### *In silico* analysis

Full length *B*. *bovis hap2* cDNA synthesized from infected female tick midgut RNA was used to compare to the complete annotated *B*. *bovis* genome sequence [5] and other apicomplexan genomes using Multiple Sequence Alignment by CLUSTALW (http://www.genome.jp/tools/clustalw/). Domain prediction of *hap2* gene was performed using the Simple Modular Architecture Research Tool (SMART) (http://smart.embl-heidelberg.de). Trans-membrane domains and signal peptides in the HAP2 protein were predicted using the Transmembrane Hidden Markov Model package 2 (TMHMM2) (http://www.cbs.dtu.dk/services/TMHMM-2.0). The detection of glycosylphosphatidylinositol (GPI) anchor was predicted using an online GPI prediction server (http://mendel.imp.ac.at/gpi/gpi_server.html).

### Polymorphism and genetic analysis

The complete gDNA sequence for *hap2* gene was compared among four geographically distinct *B*. *bovis* strains including Texas strain, Mo7, Argentina L17 virulent and Argentina L17 attenuated. Strain-specific single nucleotide polymorphisms (SNPs) were then estimated in order to calculate the ratio of synonymous to non-synonymous changes. To estimate ω (dN/dS ratio), “SNAP” was used (http://hcv.lanl.gov/content/sequence/SNAP/SNAP.html). The parameters were set up as follows: ω >1 indicated positive selection, as the selection had caused some amino acid substitution; ω<1 indicated occurrence of purifying selection and a high degree of sequence conservation [21]. Nucleotide substitutions were calculated manually.

### Knockout of the *Babesia bovis hap*2 gene

**Fig 1:**
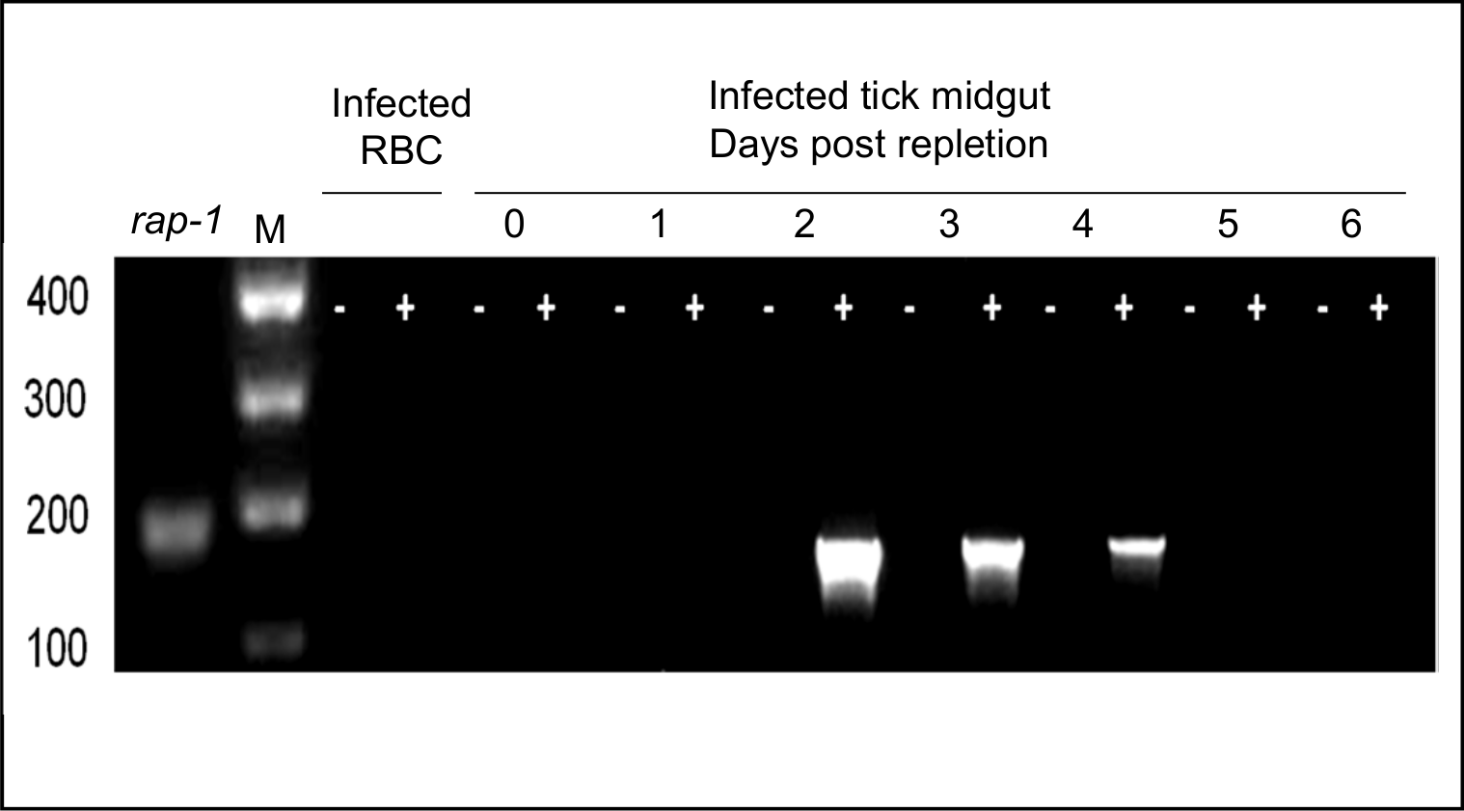
RT-PCR analysis of *B*. *bovis* hap2. Analysis was performed in *B*. *bovis* from blood from an acutely infected animal, and in dissected tick midguts of female ticks that fed on animals acutely infected with *B*. *bovis*. Tick samples were obtained at 0–6 days post-repletion. Amplifications were performed on samples with (+) or without (-) the addition of reverse transcriptase. RBC: red blood cells. *rap1*: positive control. Molecular size markers in base pairs are indicated on the left side.

*B*. *bovis* Texas strain parasites were maintained in long-term microaerophilous stationary-phase (MASP) cultures as previously described [22, 23]. Cultured blood parasites were used as to knockout the *hap2* gene. Briefly, a recombinant plasmid containing a fusion luciferase-GPF-BSD (*LUC-GFP-BSD*) gene under the control of the *ef-1α-B* promoter flanked by portions of the *hap2* gene, 660 bp in 3’ and 950 bp in 5’, was constructed (Fig 1A and 1B, GenBank accession number: KX234096).

*E*. *coli* were transformed, 5 colonies selected, and grown overnight in 5 ml of LB broth. The *phap2-luc-gfp-bsd* plasmids were extracted and submitted for sequencing. Recombinant plasmids were purified using EndoFree Plasmid Maxi Kit (Qiagen, Santa Clarita, CA) according to the manufacturer’s protocol (EndoFree Plasmid Maxi Kit, cat # 12362). Twenty μg of plasmids *phap2-luc-gfp-bsd* or pBlueScript (pBS) as a control were diluted into 25 μl Cytomix and electroporated with 75 μl of 20% *B*. *bovis* infected erythrocytes as previously described [24]. Six hours after transfection, blasticidin was added to culture medium to a final concentration of 4 μg/ml for parasite selection. Parasite growth was determined by counting the parasitemia using Giemsa stained blood smears. One week after transfection, the expression of GFP was examined by fluorescent microscopy as previously described [25].

### Evaluation of knockout *hap2* using transfected *B*. *bovis* clones

The *B*. *bovis* transfected-*hap2KO-gfp-bsd (Tf-hap2KO-gfp-bsd)* line was cloned by fluorescence activated cell sorting using 96-well plates [26].

Genomic DNA was isolated from *Tf-hap2KO-gfp-bsd* clonal line (*cln*) and *B*. *bovis* wildtype Texas strain. Briefly, *B*. *bovis Tf-hap2KO-gfp-bsd* was expanded to 25% parasitemia. The erythrocytes were pelleted and washed with phosphate-buffered saline. Erythrocytes were lysed with red blood cell lysis solution (Qiagen, Hilden, Germany) incubated for 5 min at room temperature. Parasites were lysed using cell lysis solution (Qiagen, Hilden, Germany) with 20 μg/ml of Proteinase K and incubated at 56°C for 30 min. Proteins were removed and DNA isopropanol precipitated, washed with 70% ethanol, and suspended in 100 μl of DNA hydration solution (Qiagen, Hilden, Germany).

A PacBio library was constructed using the SMRTbell Template Prep Kit v1.0. Genomic DNA was sheared using the Covaris G-Tube at 1350G for 20 min, cleaned and size selected using Ampure XP beads (Beckman Coulter, Indianapolis, IN). Standard sequencing was performed on the PacBio RSII using P6/C4 sequencing chemistry and MagBead loading. Genome sequences were assembled *de novo* with the Hierarchical Genome Assembly Process (HGAP) v 2.0 that is integrated into the SMRT analysis package. Single contig containing transfection specific sequences in the genome of the transfected clonal line were identified using BLAST utilizing all portions of the donor plasmid as queries.

### *In vitro* induction of *B*. *bovis* sexual stage parasites with xanthurenic acid

Sexual forms were induced essentially as previously described [27], with few modifications. The parasites used in these experiments were derived either from stabilates generated from blood of an infected splenectomized calf, or from *hap2*-KO culture, and maintained in *in vitro* cultures for one week before induction. These *in vitro* cultured *B*. *bovis* infected erythrocytes were suspended in an induction medium consisting of 0.465 ml final volume of culture (from a 100 ml stock solution containing 58 ml HL-1 culture medium (Lonza, Rockland, ME, USA), 40 ml bovine serum, 0.01 M TAPSO, 1 ml of 100X antibiotic-antimytotic solution (Invitrogen, CA, USA), and 100 uM xanthurenic acid (Sigma, St. Louis, MO, USA), with 10% bovine red blood cells (0.0465 ml of packed blood). Cultures were incubated at 26°C in air for up to 20h. Cultures were also incubated with the induction medium at 37°C for 20h in a MASP as previously reported [28], or in induction medium without xanthurenic acid at 26°C for 20 h.

### Polyclonal antibody production and immunoblot assay

Three synthetic peptides from the extracellular region of HAP2 were manufactured by BioSynthesis, Inc. (Texas, USA). Peptide 1: DGPEKRFRQRKGFFVC (15-mer, amino acids 2 to 17), peptide 2: KTPKGGAKKKKQKLDSSEWEHK (21-mer, amino acids 454 to 475) and Peptide 3: ERKREQESRERQAEHER (17-mer, amino acids 726 to 743). The peptides were conjugated to keyhole limpet hemocyanin (KLH) and used to immunize rabbits (BioSynthesis, Lewisville, TX, USA). Rabbits were inoculated with 0.5ml of conjugated peptides (conc.at 1.43 mg/ml) mixed in complete Freund’s adjuvant for the initial inoculation and in incomplete Freund’s adjuvant for all the booster injections. The adjuvants were mixed with the antigens at a ratio of 1:1. Inoculations were performed subcutaneously along the back, and intramuscularly in the hind limbs. All injections (less than 0.2ml/site) were done at multiple sites regardless of the route. The resulting immune sera were titrated by ELISA and used in subsequent immunoblot assays. The specificity of the anti HAP2 polyclonal antibody was further tested in immunoblots using non-purified recombinant HAP2 expressed in prokaryotic expression system pBAD/thio-TOPO (Invitrogen, CA, USA) (S1 Fig). The full size *hap2* gene was amplified from *B*. *bovis* cDNA by PCR amplification, using primers *hap2* full length (Table 1). The resulting amplicons were cloned into the pBAD/thio-TOPO vector. Plasmid DNA extracted from *E coli* positive clones were sequenced to confirm their identity and the correct orientation of the *hap2* insert. One positive clone was selected for expression of HAP2 in *E*. *coli*-transformed cultures (125 ml) using expression induction with 0.2% arabinose for 3 h at 37°C. Bacterial pellets were suspended and homogenized in lysis buffer-Nonidet-P40 (NP-40) (150 mM sodium chloride 1.0% NP-40–50 mMTris, pH 8.0) and protease inhibitor (1 μg/ml). Total protein from cell lysate used for the immunoblots. The polyclonal antibody for Bbo 6-Cys A was described and obtained in a previous study [16].

The antigens used in the immunoblots were prepared from *B*. *bovis*-infected erythrocyte culture or xanthurenic acid induced culture. Sexual stages were pelleted from induced culture by differential centrifugation of 400xg to 2,000xg to pellet the sexual stages. Parasites were suspended and homogenized in lysis buffer and 1 μg/ml protease inhibitor (Roche Diagnostics, Indianapolis, IN, USA). Total protein was quantified by Micro BCA Protein Assay (Thermo Fisher Scientific Inc., Waltham, MA, USA), 5 μg of total protein were mixed with 5x SDS-PAGE sample buffer (GenScript, Fl, USA), boiled for 5 min and then sonicated for 2 min with 20 sec intervals, and separated into 4–20% Mini-PROTEAN TGX Precast Gels (BioRad Laboratories, Hercules, CA, USA). Proteins were transferred to a nitrocellulose membrane (Whatman, Dassel, Germany) for 1 h at 100 V. The membranes were blocked with 5% skim milk in TBS (Tris-buffered saline: 50 mM Tris-HCl/ 150 mM NaCl, pH7.6) for 1 h at room temperature, washed three times in TBS and incubated for 1 h with 1:100 dilution of primary antibody against *B*. *bovis* 6-Cys A and HAP2. Monoclonal RAP-1 antibodies were used to detect RAP-1 protein during *in vitro* cultured *B*. *bovis* [29] as well as pre-immune rabbit serum as positive and negative controls, respectively. After three washes in TBS, The membranes were incubated for 30 min with 1:5000 dilution of HRP conjugated goat anti-rabbit IgG (H+L) or anti-mouse IgG (H+L) antibodies (KPL, Gaithersburg, Maryland, USA), and washed again three times with TBS. Antibody reactivity was visualized using chemiluminescent HRP antibody detection reagents (KPL, Gaithersburg, Maryland, USA).

### Detection of surface exposed proteins on *B*. *bovis* sexual stages

Sexual stages were enriched from *in vitro* cultures induced by 20h using differential centrifugation as described above. Parasites were washed in 3% normal goat serum in PBS. A portion of the cells were then incubated for 1h with 1:50 anti-HAP2, or anti-6-Cys A primary antibodies diluted with 10% normal goat serum in PBS. The cells were then washed twice in the PBS by 400 *xg* centrifugation and incubated for 30 min with 1:1000 goat anti-rabbit Alexa Fluor 555 secondary antibodies (Thermo Fisher Scientific) diluted with 10% normal goat serum. The cells were again washed twice with PBS, and air dried on slides, and nuclei were stained with 4, 6-Diamidino-2-phenylindole dihydrochloride (Thermo Fisher Scientific). Identically produced negative controls were performed using pre-immune (PI) rabbit serum instead of the primary antibodies. All samples were independently visualized by fluorescent microscopy and images were processed as described below. Slides were viewed and digitally photographed using an Axio Imager, M1 microscope (Carl Zeiss Imaging, Inc., Phoenix, AZ, USA). The microscope is equipped with an X-Cite 120 Fl illuminating system (EXFO Photonic Solutions). Digital images were captured using an AxioCam MRm digital camera connected to a desktop computer running the AxioVision (version 4.8.1.0) program. Image stacks were obtained using optimal z-axis spacing [250 nm z-step, Plan-Apochromat 63x/1.4 oil M27 objective (Carl Zeiss Imaging, Inc., Phoenix, AZ, USA)]. Z-stack image files were imported for processing into the ImageJ-based open source processing package Fiji (version 1.48b; http://pacific.mpi-cbg.de/) [30]. Surface exposure of HAP2 in *in vitro* Xanthurenic acid induced parasites was confirmed by analyzing parasites in IFA after trypsinization [31] as follows. Sexual stages of *B*. *bovis* induced in *in vitro* cultures were washed twice in PBS by 400 *xg* centrifugation. Cells were trypsinized for 30 minutes at 37^o^ C with 0.05% trypsin-EDTA (Gibco BRL/Invitrogen, Carlsbad, CA, USA), trypsinization was terminated with the addition of trypsin inhibitor (Sigma-Aldrich, St Louis, MO, USA) for 10 min at 37°C. Parasites were then washed in 3% normal goat serum in PBS. A portion of the cells were then incubated for 1h with 1:50 anti-HAP2 primary antibodies diluted with 10% normal goat serum in PBS, and washed twice in the PBS by 400 *xg* centrifugation and incubated for 30 min with 1:1000 goat anti-rabbit Alexa Fluor 555 secondary antibodies (Thermo Fisher Scientific, CA, USA) diluted with 10% normal goat serum. The cells were again washed twice with PBS. To estimate cell viability, cells were suspended in PBS and mixed with equal volume of 6-Carboxyfluorescein Diacetate (6-CFDA [31], final concentration in PBS. 10 μg/ml: Calbiochem-Behring, La Jolla, CA, USA), and Incubated at room temperature for 15 minutes. The cells were then washed once with PBS and incubated with nucleic acid stain Hoechst 33342 (Thermo Fisher Scientific, CA, USA) for 30 minutes. Finally, cells were washed twice with PBS, and air dried on slides. All samples were independently visualized by fluorescent microscopy as described above.

### Ethics statement

This study was approved by the Institutional Animal Care and Use Protocol Committees of the University of Idaho, Moscow, Idaho (protocol #2016–20) in accordance with institutional guidelines based on the U.S. National Institutes of Health (NIH) Guide for the Care and Use of Laboratory Animals. The rabbit antibodies were generated according to the approved animal care protocol D16-00398 (OLAW) by BioSynthesis, and to USDA Research license number 23-R-0089.

## Results

### The multi-intron, single copy, *B*. *bovis hap2* gene encodes for a ~84 kDa cell membrane protein

A single copy *B*. *bovis hap2* gene is located in between 1,452,162 bp and 1,454,808 bp of chromosome 3, containing 8 introns and 9 exons. This multi-intron structure is usually conserved in the *hap2* genes among apicomplexan parasites [32–35]. The annotated *hap2* mRNA [GenBank XM_001611756] revealed an orf of 2,271 bp, coding for a 79.53 kDa protein containing 723 amino acids. *B*. *bovis* HAP2 protein contains a single HAP2 domain Similar to *Plasmodium falciparum* 7G8 HAP2 (XP_001347424).This domain is functionally involved in a highly conserved sperm protein that is essential for gamete fusion. The HAP2 domain is located between amino acids 348 and 394, suggesting a similar conserved function for this gene among these parasites. The HAP2 domain is predicted to be located in the extracellular region of the protein, which is likely exposed on the surface of the parasites.

Overall, *B*. *bovis* HAP2 deduced amino acid sequence appears relatively well conserved when compared with its homologues in other species. *In silico* predictions suggests that the *B*. *bovis* HAP2 protein lacks a glycosyl phosphatidylinositol (GPI) anchor. In addition, HAP2 is also predicted to contain a signal peptide between amino acids 1–33, a hydrophobic transmembrane domain located between amino acids 683–705, and a predicted coiled coil domain between amino acids 721–753, located near the C-terminus. The coiled coil domain is also present in an identical location in many viral fusion proteins, consistent with possible role in the membrane fusion reaction. Collectively, all these features are suggestive of the possible trafficking of HAP2 to the external surface of the parasite, and its possible role as a fusogenic protein.

### The *hap2* gene is highly conserved among *B*. *bovis* strains

We examined the occurrence of sequence variation and single nucleotide polymorphisms (SNPs) among the *hap2* gene among distinct *B*. *bovis* strains. The *hap2* gene is highly conserved among the distinct strains (99% to 99.9% aa identity). The analysis was performed using a sequence database including *hap2* gene derived from *B*. *bovis* Texas strain, Mo7, Argentina L17 virulent and Argentina L17 attenuated. The calculated synonymous and non-synonymous S/N ratios (Table 2) with the parameter, ω, (ω = dN/dS), as an indicator of potential selection pressures. In all cases, ω of less than 1 was obtained and revealed no evidence for positive selection for the *hap2* gene, suggesting that *B*. *bovis hap2* gene is under no diversifying immune selection. The data also indicates a low likelihood of selective forces such as immune pressure of the host acting on the evolutionary history of this gene, consistent with low or lack of exposure of the protein to the immune system of the host during infection, which suggests no expression of *hap2* in blood stages of the parasite.

**Table 2:** Polymorphism and average SNPs of the *hap2* gene among different distinct *B*. *bovis* strains.

	Texas	Mo7	Argentina L17 virulent	Argentina L17 attenuated
Nucleotide substitutions	21	28	20	27
Synonymous substitutions	9	10	8	10
Non-synonymous substitutions	12	18	12	17
Average dN	0.0039	0.0044	0.0035	0.0044
Average dS	0.0052	0.0079	0.0052	0.0074
ω (dN/dS)	0.75	0.55	0.67	0.59

dN: non-neutral polymorphism divergence; dS: neutral divergence.

### The *hap2* gene is differentially transcribed in tick stages of *B*. *bovis*

The pattern of *hap2* transcript expression was investigated by RT-PCR analysis performed on RNA extracted from *B*. *bovis* infected erythrocytes and tick midguts. Previous transcriptome and RNA sequence analysis using short-term cultured merozoites from strains differing in origin and virulence phenotypes show that the *hap2* gene is transcribed at very low or undetectable levels compared to constitutively expressed *rap1* in the blood stages (S2 Fig) [36]. Consistently, only *rap-1*, but not *hap2* transcripts were detected in *B*. *bovis* blood stages by RT-PCR. In contrast, *hap2* transcripts were transiently detected at days 2, 3 and 4, but not at days 0, 1, 5 and 6 post-repletion during the development of *B*. *bovis* in the tick midgut (Fig 1). Sequence analysis of the RT-PCR derived 165 bp amplicon (Fig 1) demonstrated identity to the *hap2* sequence from the annotated *B*. *bovis* genome [5]. Overall, the results indicate that the *hap2* gene is differentially transcribed during *B*. *bovis* development within tick midgut, but not during development of parasites within the mammalian host. Interestingly, the differential intensity of the RT-PCR bands (Fig 1) suggests that expression of *hap2* peaks at day 2 post repletion, but this observation needs to be confirmed using a quantitative assay.

### Disruption of the *hap2* gene does not affect the development of *in vitro* cultured *B*. *bovis* blood stages

**Fig 2:**
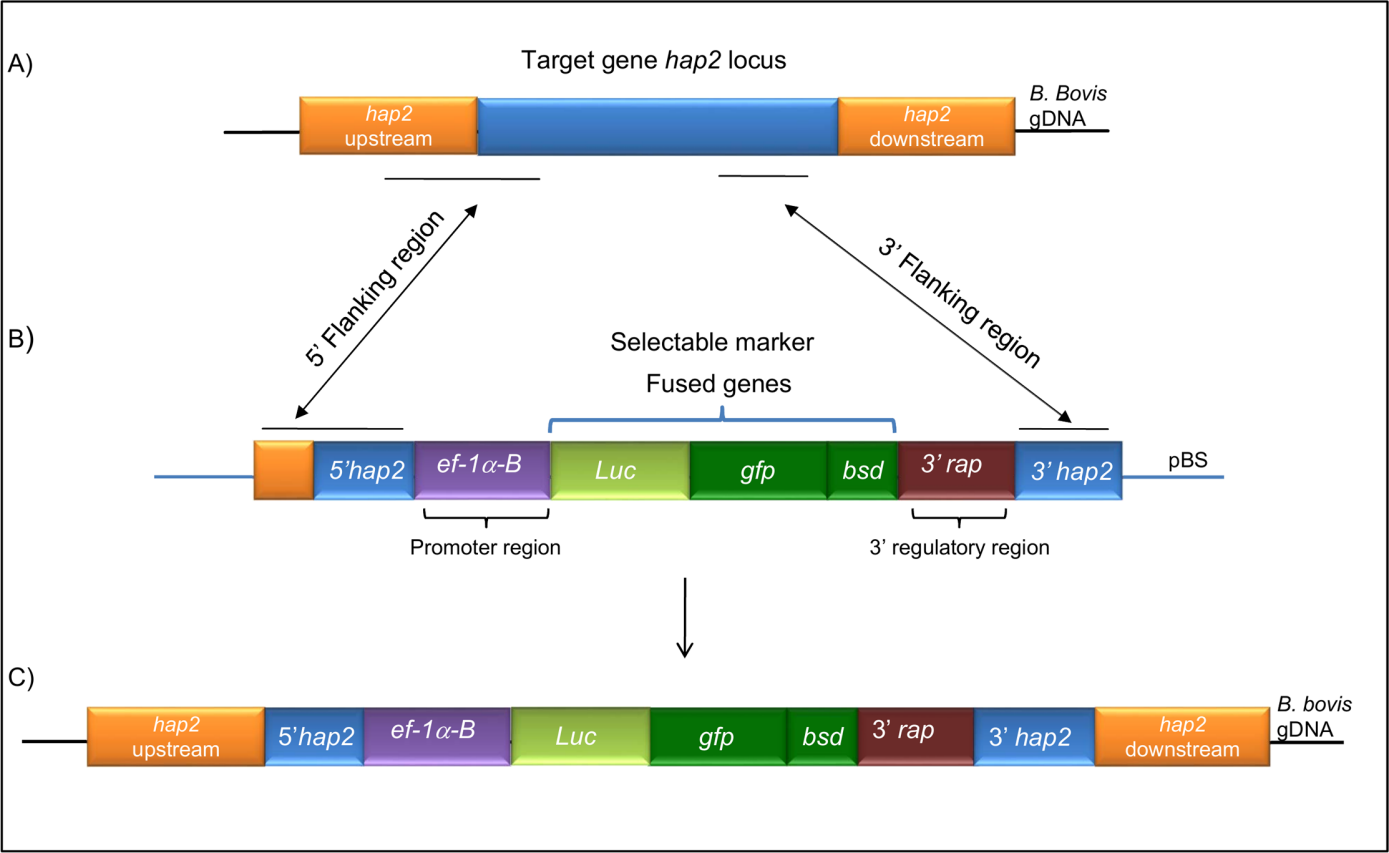
Schematic diagrams showing. **A.** Structure of the targeted *hap2* gene locus; **B.** Organization of the plasmid *phap2-lucgfpbsd* used to disrupt the *B*. *bovis hap2* gene; **C.** Deduced structure of the disrupted *hap2* gene locus in the transfected clonal line*Tf-hap2KO-gfp-bsd*.

The *hap2* gene was disrupted using the transfection plasmid *phap2-lucgfpbsd*. The structure of the *B*. *bovis hap2* locus and the experimental design for the disruption of *hap2* are represented in Fig 2.

**Fig 3:**
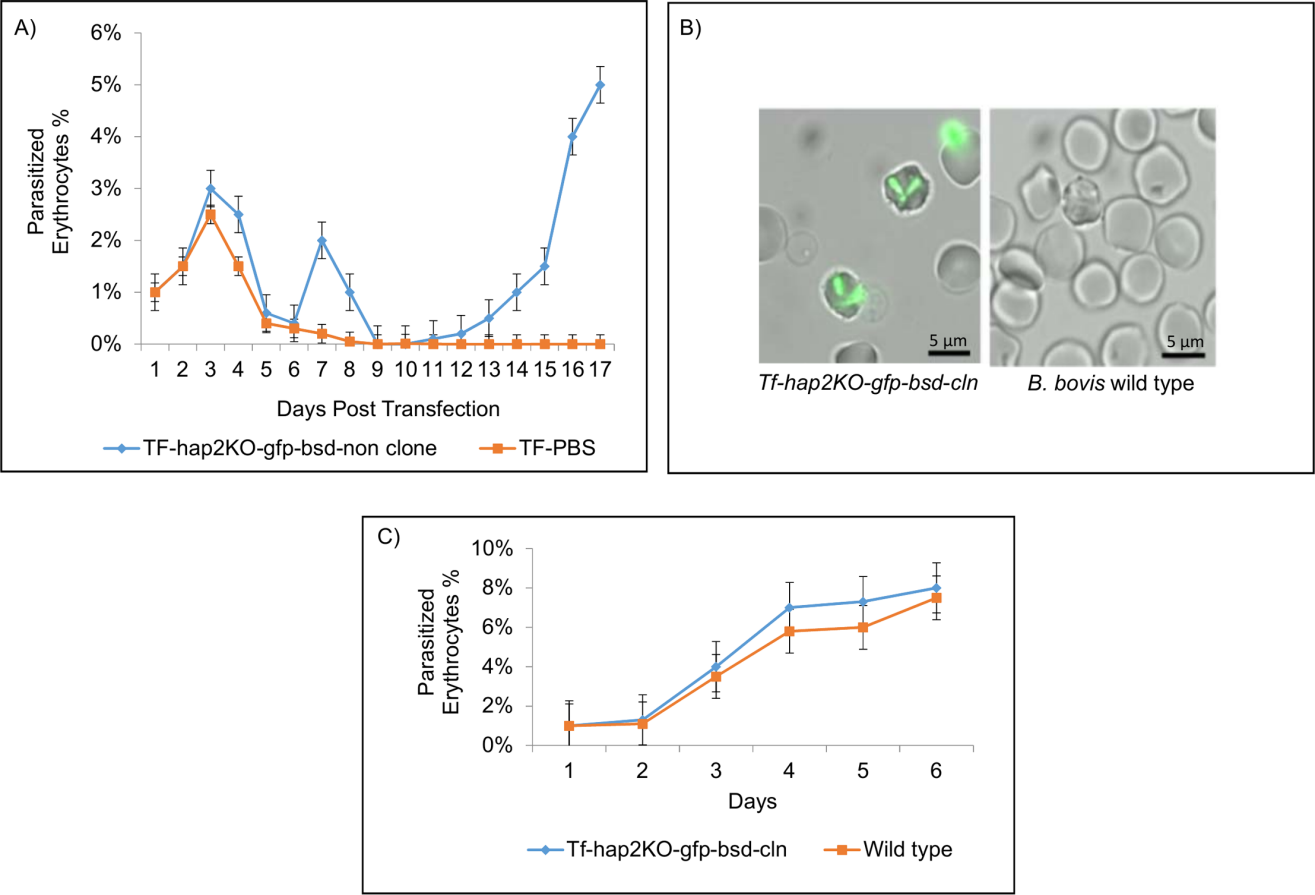
Disruption of the *B*. *bovis hap2* gene and cloning and phenotypic characterization of a *hap2* KO strain. **A.** Growth curve describing the kinetics of emergence of the transfected parasite line *Tf-hap2KO-gfp-bsd* upon blasticidin selection. **B.** Detection of GFP expression by the transfected *B*. *bovis* clonal line *Tf-hap2KO-gfp-bsd*-*cln* derived from the parasite line *Tf-hap2KO-gfp-bsd*, left panel is *Tf-hap2KO-gfp-bsd*-*cln* and right panel is the non-transfected *B*. *bovis* wild type strain T3B. Scale bar: 5 μm. **C.** Growth curves of the *B*. *bovis Tf-hap2KO-gfp-bsd-cln* clonal line and non-transfected *B*. *bovis* strain T3B parasite line (wild type) showing similar replication kinetics *in vitro* without blasticidin selection. The “X” axis represents the percentage of infected erythrocytes in *in vitro* cultures and the “Y” axis the days after the initiation of the *in vitro* cultures.

Selection with blasticidin resulted in the emergence of the transfected and green fluorescent *Tf-hap2KO-gfp-bsd* cell line. In contrast, parasites electroporated with the control *pTf-pBS* plasmid did not survive upon blasticidin selection (Fig 3A). Clonal cell lines were generated from the mixed parasite line *Tf-hap2KO-gfp-bsd* by flow cell sorting [37, 38]. Clonal lines were evaluated by expression of GFP (Fig 3B). The clonal line termed *Tf-hap2KO-gfp-bsd-cln* was selected for further analysis. Growth curve analysis demonstrated that both *B bovis Tf-hap2KO-gfp-bsd-cln* and its wild type parental strain had similar replication kinetics (Fig 3C).

The clonal line *Tf-hap2KO-gfp-bsd-cln* was fully sequenced. Analysis of the full genomic DNA sequence of the *Tf-hap2KO-gfp-bsd-cln* line revealed an output of polished assembly of 609 contigs with the largest contig being 87kb. BLAST of the *hap2* gene (BBOV_III006770) against assembly contigs revealed a single hit at contig 1592. The area covered by contig 1592 was roughly 1,438,762 to 1,455,743 of chromosome 3, which contained a ~6, 448 bp insertion (GenBank accession number: KX234097). Full sequencing of the genome of the *Tf-hap2KO-gfp-bsd-cln* confirmed replacement of the *hap2* gene by the 5’ *hap2* (935 bp), *EF* promoter (761 bp), luciferase (1,651 bp), *gfp-bsd* (1,100 bp), 3’ *rap1* (1,288 bp), and 3’ *hap2* (656 bp) fragments, present in the transfection plasmid. Thus, analysis of the structure of the *hap2*-KO gene in the clonal line *Tf-hap2KO-gfp-bsd-cln* indicates that these sequences were inserted by homologous recombination. It was the only foreign DNA insert detected by whole genome sequencing. The rest of the *B*. *bovis Tf-hap2KO-gfp-bsd*-*cln* genomic sequence was essentially identical to the wildtype *B*. *bovis* genome sequence [5]. Collectively, these data confirmed a successful insertion of the transfected genes disrupting the targeted *hap2* locus of *B*. *bovis*, and suggests that disruption of the *hap2* locus did not affect the pattern of growth of the parasite in *in vitro* cultures.

### The *hap2* gene is expressed during *in vitro* induction of *B*. *bovis* sexual stages

**Fig 4:**
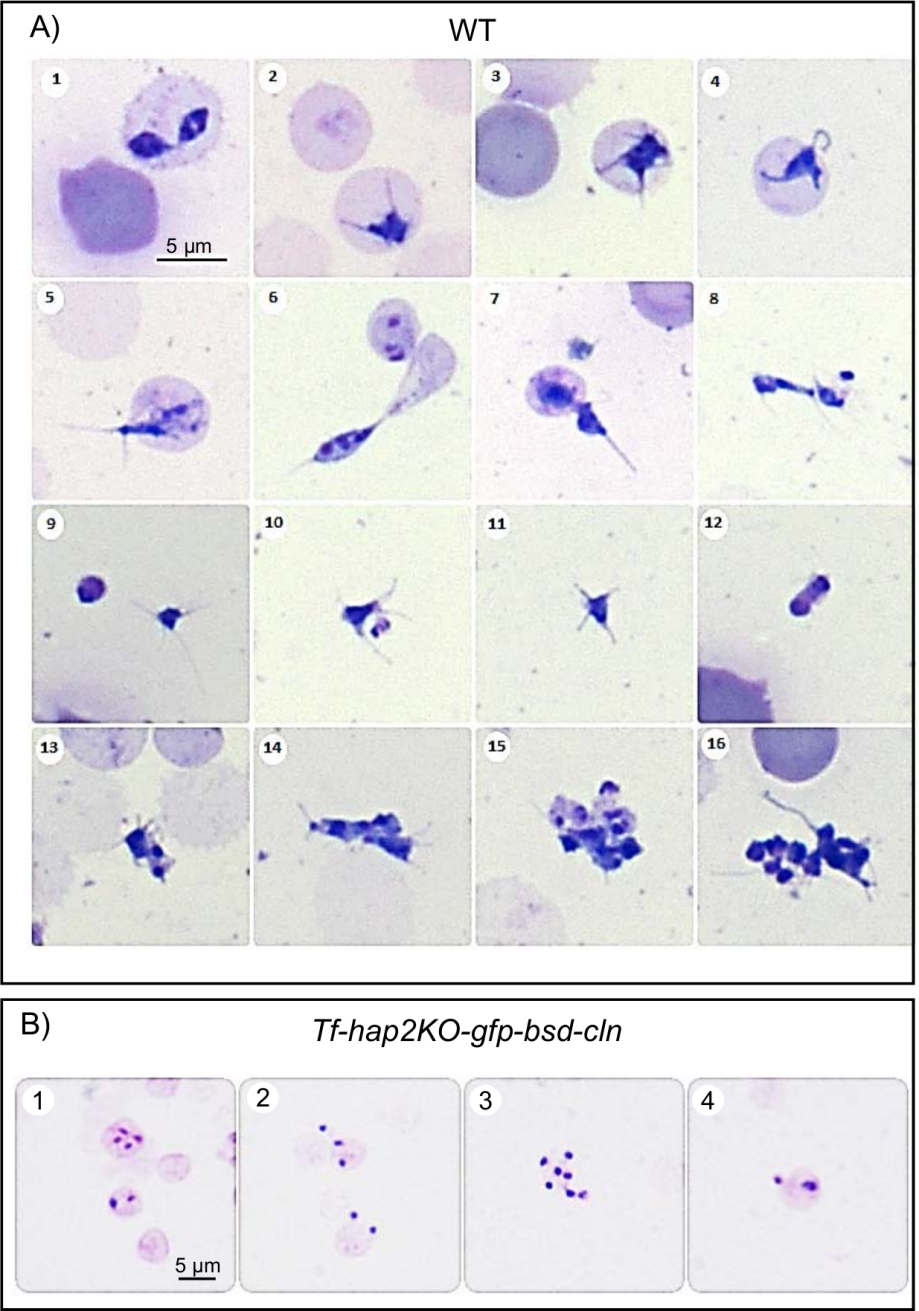
Photomicrograph of Giemsa-stained *B*. *bovis* smears. **A.** Morphological changes of *B*. *bovis* wild type parasites developing in induced *in vitro* cultures, (1–4) lntraerythrocytic parasites, develop ray bodies (Strahlenkorper) whilst still intracellular. (5–7) Strahlenkorper egressing from erythrocytes. (8–11) Polymorphic multi-nucleated population of Strahlenkorper with long and short spikes. (12, 13) Two strahlenkorper adhering to each other along cell membranes. (14–16) Large aggregates of adhering multi-nucleated Strahlenkorper, occurring 20 h after induction. **B.** *B*. *bovis Tf-hap2KO-gfp-bsd-cln* parasites with induction medium (XA) at 26°C (1–4). Bars, 5 μm.

*B*. *bovis* sexual stages were induced *in vitro* by decreasing the temperature to 26°C and the addition of xanthurenic acid to the culture media. Microscopic inspection of Giemsa stained cells from induced cultures showed the presence of extra-erythrocytic parasites with long projections and large round parasite stages, indicative of parasite sexual stage development (Fig 4A). No such sexual stages forms were found upon similar microscopic inspection of *Tf-hap2KO-gfp-bsd-cln* parasites (Fig 4B), and fluorescent microscopy inspection of *Tf-hap2KO-gfp-bsd-cln* live parasites (S3 Fig) developing in *in vitro* cultures with induction medium xanthurenic acid (XA) at 26°C.

**Fig 5:**
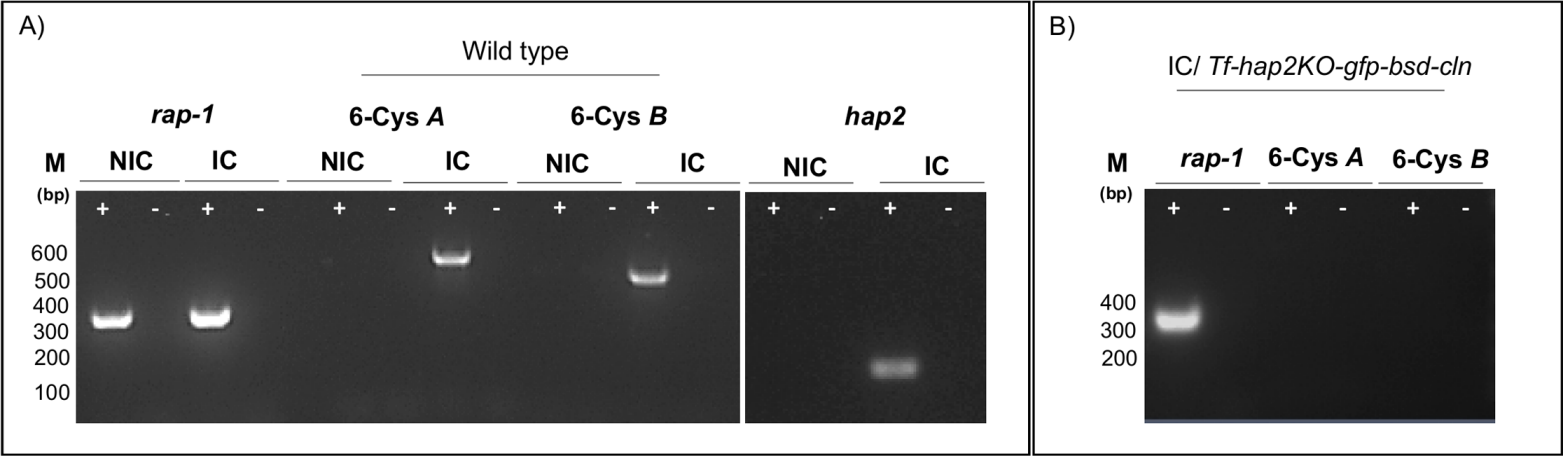
Transcription analysis of *B*. *bovis* blood stages and induced sexual stages. **A.** RT-PCR analysis for the detection of 6-Cys *A*, 6-Cys *B* and *hap2* transcripts in wild type *B*. *bovis* parasites. The RT-PCR analysis was performed using non-induced (NIC) and induced (IC) *in vitro* cultures. **B.** 6-Cys *A*, 6-Cys *B* RT-PCR products were not detectable in *Tf-hap2KO-gfp-bsd-cln* induced parasites. Amplifications were performed on samples with (+) or without (-) the addition of reverse transcriptase. RT-PCR amplification of *rap1* was used as a positive control. M represents the sizes of molecular markers in base pairs. **NIC:** non-induced culture, **IC:** induced culture.

Previous comparative studies performed in *B*. *bovis* parasites from *in vitro* cultures and in tick midgut, defined the expression of the 6-Cys *A* and *B* genes as markers of sexual stage parasites [16]. A similar comparative transcript analysis using RT-PCR and sequencing confirmed expression of 6-Cys *A* and *B* and *hap2* genes in parasites emerging upon *in vitro* sexual stage induction. In contrast non-induced parasites failed to produce *hap2* and 6-cys *A* and *B* transcripts (Fig 5A). In addition, *Tf-hap2KO-gfp-bsd-cln* parasites, produced *rap-1* transcript, but failed to produce *6-cys A* and *B* transcripts (Fig 5B) upon xanthurenic acid induction.

**Fig 6:**
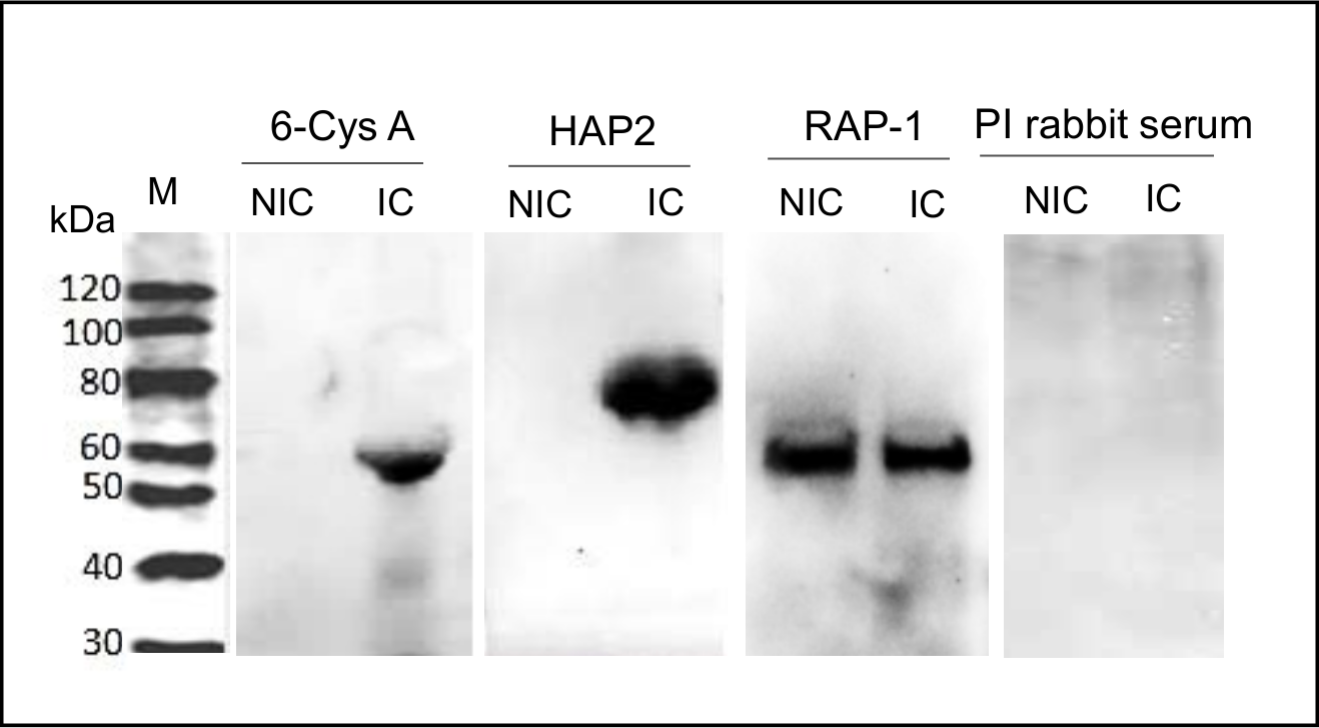
Western blot analysis using antibodies against 6-Cys A, and HAP2, performed on non-induced (NIC) and induced (IC) *B*. *bovis* parasites developed in *in vitro* cultures. Monoclonal RAP-1 antibodies were used to detect RAP-1 protein during *in vitro* cultured *B*. *bovis* as a positive control, and pre-immune rabbit serum as a negative control PI rabbit serum. Size markers (M) in kDa are indicated at the left side.

In addition, anti-6-Cys A and anti-HAP2 antibodies react with wild type *B*. *bovis* antigens of ~60 kDa and~80 kDa respectively in induced cultures but did not recognize any native protein in non-induced *B*. *bovis* culture in immunoblots (Fig 6). In contrast, the control 60 kDa RAP-1 is recognized in lysates from both, induced and non-induced parasites with comparable signal intensities (Fig 6). *Tf-hap2KO-gfp-bsd-cln* Induced parasites didn’t show reactivity against anti-HAP2 antibodies (S4 Fig). The size of the antigens recognized by all antibodies matches the predicted sizes of the RAP-1, 6-Cys A and anti-HAP2 proteins. Thus, the data is consistent with co-expression of the sexual stage marker 6-Cys A and HAP2 proteins in induced wild type *B*. *bovis* cultures, but not in non-induced cultures.

**Fig 7:**
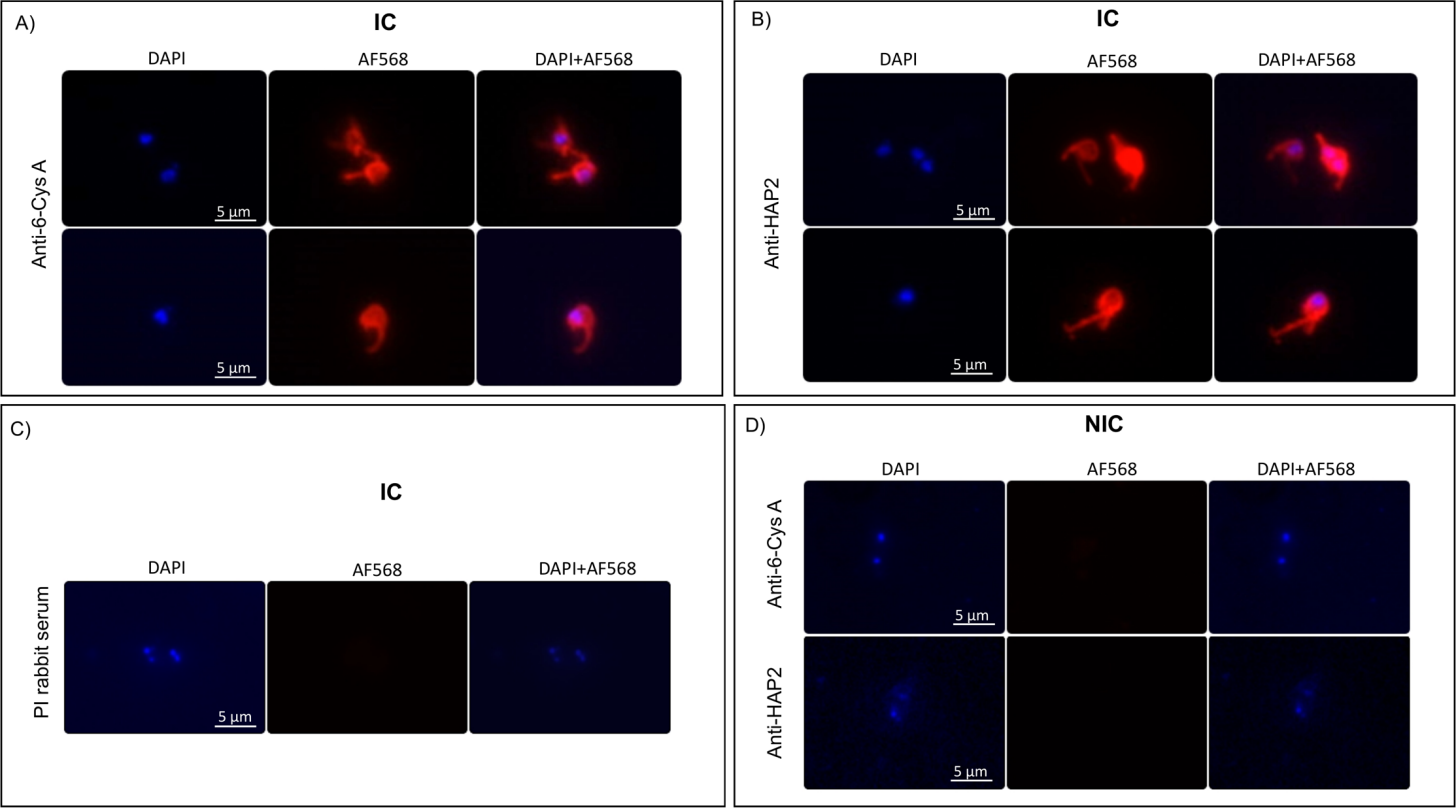
Live immunofluorescence analysis for the expression of 6-Cys A and HAP2 in the surface of induced *B*. *bovis* extracellular parasites. **A.** *B*. *bovis* induced cells incubated with anti-6-Cys A and goat anti-rabbit tagged with Alexa Fluor 555 and stained with DAPI; **B.** *B*. *bovis* induced cells incubated anti-HAP2 and goat anti-rabbit tagged with Alexa Fluor 555 and stained with DAPI; **C.** Negative control preimmune (PI) rabbit serum as primary antibody, and stained with DAPI; **D.** non-induced *B*. *bovis* cells Incubated with Anti 6-Cys A and Anti-HAP2 and goat anti-rabbit tagged with Alexa Fluor 555 and stained with DAP. **NIC:** non-induced culture, **IC:** induced culture, Bars, 5 μm.

In addition, live immunofluorescence assays (Live IFA) confirmed expression of the 6-Cys A and HAP2 proteins on the surface of sexual stage induced *B*. *bovis* T3B strain, but not in the non-induced parasites (Fig 7A–7D). Overall the data is consistent with the notion that expression of the *hap2* gene is associated with the development of *B*. *bovis* sexual stage forms induced *in vitro*.

**Fig 8:**
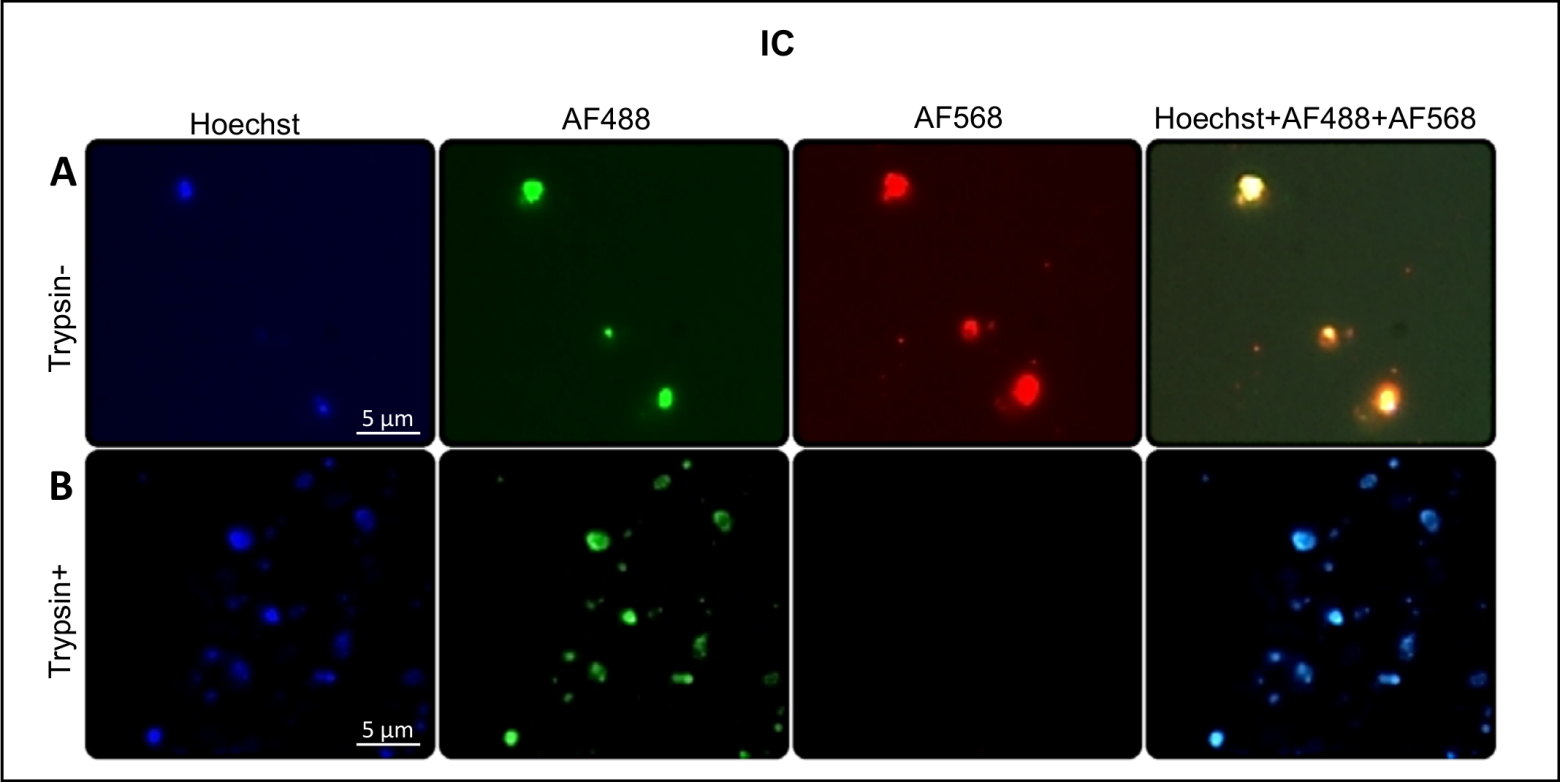
HAP2 expression in the surface of *B*. *bovis* induced parasites. **A.** *B*. *bovis* induced cells incubated with HAP2 and goat anti-rabbit tagged with Alexa Fluor 555 and stained with 6-CFDA and nucleic acid stain Hoechst 33342; **B.** *B*. *bovis* induced cells treated with trypsin and incubated with anti-HAP2 and goat anti-rabbit tagged with Alexa Fluor 555 and stained with 6-CFDA and nucleic acid stain Hoechst 33342; IC: induced culture, Bars, 5 μm.

We confirmed surface exposure of HAP2 by performing live immunofluorescence analysis on trypsin-treated *B*. *bovis* induced cells (Fig 8). Parasites treated with trypsin are no longer recognized by anti-HAP2 antibodies in live immunofluorescence assays (Fig 8 AF 568). In addition, the trypsin treatment did not alter the membrane integrity and the viability of the treated parasites, as they are still stained with the vital 6-CFDA stain in a pattern that is similar to non-trypsin-treated parasites (Fig 8. AF 488).

## Discussion

The *hap2* gene products in *B*. *bovis* related apicomplexan parasites have been consistently associated with differential expression and the formation of sexual forms. In this study, we demonstrated transcription of *hap2* in parasites residing in the midgut of replete *R*. *microplus* female tick fed on a bovine infected with *B*. *bovis*, but not in *in vitro* cultured blood stages. A requirement of *B*. *bovis* to perpetuate its life cycle is the ability to develop sexual stages within the lumen of the *Rhiphicephalus* tick midgut. The fusion of gametes results in a stage infectious to tick midgut epithelial cells. Within midgut cells, *B*. *bovis* transforms into kinetes. This stage egresses from the midgut into the hemolymph to further infect ovaries [12]. Previous work indicated the differential expression of members of the 6-Cys family in tick stages [16], suggested that specific *B*. *bovis* proteins are necessary for parasite development within the tick vector. Interestingly, transcription of *hap2* is limited to days 2 to 4 after dropping. This pattern of transcription may be required for synchronized sexual stages formation, or be related to the timing of gamete fusion inside the tick midgut. These observations are consistent with *Plasmodium* where HAP2 is expressed only in gametocytes and gametes [15]. Differential expression of HAP2 is indispensable for fertilization of *Plasmodium* parasites, with a demonstrated specific fusion function during gamete interaction [15].

Importantly, genome sequence analysis among *B*. *bovis* isolates demonstrated that HAP2 is highly conserved with an identity of 99% to 99.9%.The high degree of HAP2 sequence conservation among strains also supports the usefulness of HAP2 as a potential antigen for vaccine development aimed to block *B*. *bovis* transmission. The synonymous and non-synonymous ratios (Table 2) revealed no evidence for positive selection for the *hap*2 gene, consistent with a low frequency of single nucleotide polymorphisms in *hap2* from different *B*. *bovis* isolates. These results suggest that *B*. *bovis* HAP2 is under no diversifying selection, a property shared with current transmission-blocking vaccine candidates in *Plasmodium* [39]. The data also suggest the occurrence of functional restrictions to sequence variations for this gene, which enhances its potential as a vaccine candidate.

We also examined if knocking out the *hap2* gene affected the growth fitness of the parasite in *in vitro* cultures. The *in vitro* growth fitness of the *hap*2 KO parasites was similar to the wildtype strain indicating that the gene is not critical for *B*. *bovis* development within erythrocytes. Importantly, full sequence of *hap*2 knocked out parasites demonstrated the insertion of a single copy of the transfected selectable marker/reporter genes disrupting the *hap*2 locus, and thus such transfected parasites are ideally suited for exploring the functional significance of the *hap2* gene in *B*. *bovis*. Furthermore, the remainder of the genome of the transfected *B*. *bovis* genome was unaltered, and no other insertions derived from the transfection plasmid were found in the genome of the KO strain confirming the high specificity and efficiency of the homologous recombination mechanisms operating in *B*. *bovis*. These data also confirm the usefulness of transfection as an approach to study gene function by disrupting gene expression in different *B*. *bovis* stages.

In this study we were able to induce *B*. *bovis* sexual forms using xanthurenic acid in *in vitro* cultured parasites for the first time. Xanthurenic acid is a metabolic intermediate derived from the metabolism of tryptophan which is present in the gut of the *Anopheles* mosquito where it is known to induce gametogenesis of *Plasmodium falciparum* [40, 41]. It remains unknown whether this metabolite is also present in the tick midguts, or if gametogenesis in *Babesia* parasites also requires a xanthurenic acid depending mechanism. However, similar to previous observations in *B*. *bigemina* [27], we were also able to induce changes in *B*. *bovis* morphology using particular culture incubation settings in the presence of xanthurenic acid. The induced parasites present several distinct morphology and shapes, consistent with previous similar inductions on *B*. *bigemina* and with forms found in the midgut of ticks engorged on *Babesia* infected cattle. Importantly, because expression of the 6-cys *A* and *B* genes are known to be markers of *B*. *bovis* sexual stages, the molecular data on expression of these two genes upon induction included in our study validated for the first time the observation that the addition of xanthurenic acid concomitant with decreased incubation temperatures, results in the induction of sexual stages, as visualized before just by changes in the morphology. In addition, and consistent with morphology changes, induction result in their progression into a life stage that is morphologically and molecularly different than the life stage forms that typical in blood parasites cultured under standard (non-induced) culture conditions. Indeed the detection of expression of the 6-cys *A* and *B* genes in these induced forms is fully consistent with the formation of sexual forms normally induced in the midgut of *R*. *microplus* ticks feeding in *Babesia* infected animals [16]. Interestingly, our data show a correlation among the inability of *Tf-hap2KO-gfp-bsd-cln* parasites to change morphology, and to generate sexual stage specific expression products such as the members of the 6-Cys gene family. In contrast, these mutant parasites *Tf-hap2KO-gfp-bsd-cln* are fully able to develop and grow *in vitro* in erythrocytes, supporting the concept that an intact copy of the *hap2* gene is required for sexual stage induction but irrelevant for blood stage development. The results can be compared with similar previous findings in malaria parasites, where sexual stage fusion was found dependent on the expression of the *hap2* gene [42–44]. Importantly, two distinct lines of evidence, direct live immunofluorescence and loss of recognition of surface exposed HAP2 upon trypsinization, supports that *B*. *bovis* induced parasites express HAP2 in their surface. Therefore, it is likely that the *B*. *bovis* HAP2 indeed also functions as an ancestral gamete fusogen in this parasite, since highly diverse eukaryotic gametes carrying loss-of-function mutations in HAP2 also fail to fuse [45].

In summary, HAP2 is differentially expressed by *B*. *bovis* during its development within *R*. *microplus* and the *in vitro* induction data suggests that surface exposed expression of this protein might be connected to the completion of the *B*. *bovis* life cycle during parasite development in the tick midgut. The absence of detectable *hap2* transcripts by *B*. *bovis* blood stages suggested that *hap2* is unnecessary for parasite development during infection of mammalian host. In contrast, the data supports that expression of HAP2 occurs in concurrence with the development of sexual stages upon induction with xanthurenic acid under *in vitro* culture conditions. Overall, these findings strongly suggest a role of *hap2* during tick stages of the parasite, probably including sexual reproduction and supports HAP2 as a leading candidate for a transmission blocking vaccine against bovine babesiosis. Further *in vivo* studies are necessary to determine if disrupting *hap2* interferes with the development of *B*. *bovis* within tick midgut and beyond.

## Supporting information

S1 Fig Western blot analysis using antibodies against a HAP2 synthetic peptide reacted against the recombinant version of HAP2. Lane 1: Bacterial lysate derived from arabinose induced recombinant bacteria; Lane 2: Bacterial lysate derived from non-arabinose induced recombinant bacteria. Size markers (M) in kDa are indicated at the left side.
(TIF)

S2 Fig Transcriptional analysis of *hap2* and *rap1* of *B. bovis* cultured blood stages. **A.** Microarray analysis of the virulent and attenuated parasites derived from *B*. *bovis* T2B strain. The Y axis indicates relative transcriptional levels. The X axis represents the name of the parasite strain. **B.** RNA seq analysis performed on the virulent and attenuated parasites derived from *B*. *bovis* L17 strain. The Y axis indicates relative transcriptional levels. The X axis represents the name of the parasite strain.
(TIF)

S3 Fig Green fluorescent parasites from *Tf-hap2KO-gfp-bsd-cln* xanthurenic acid-induced culture at 26°C, nuclei stained with Hoechst. (TIF)

S4 Fig Western blot analysis using antibodies against HAP2, performed on Lane 1: induced (IC) *B. bovis* wild type parasites; Lane 2: induced (IC) *B. bovis Tf-hap2KO-gfp-bsd-cln* parasites developed in *in vitro* cultures. Monoclonal RAP-1 antibodies were used to detect *B*. *bovis* RAP-1 protein as a positive control. Size markers (M) in kDa are indicated at the left side.
(TIF)
