# Supplementary figures and images for: The Babesia bovis hap2 gene is not required for blood stage replication, but expressed upon in vitro sexual stage induction
Source: PLoS Negl Trop Dis. 2017 Oct 6;11(10):e0005965. doi: 10.1371/journal.pntd.0005965 (PMC5646870; doi:10.1371/journal.pntd.0005965)

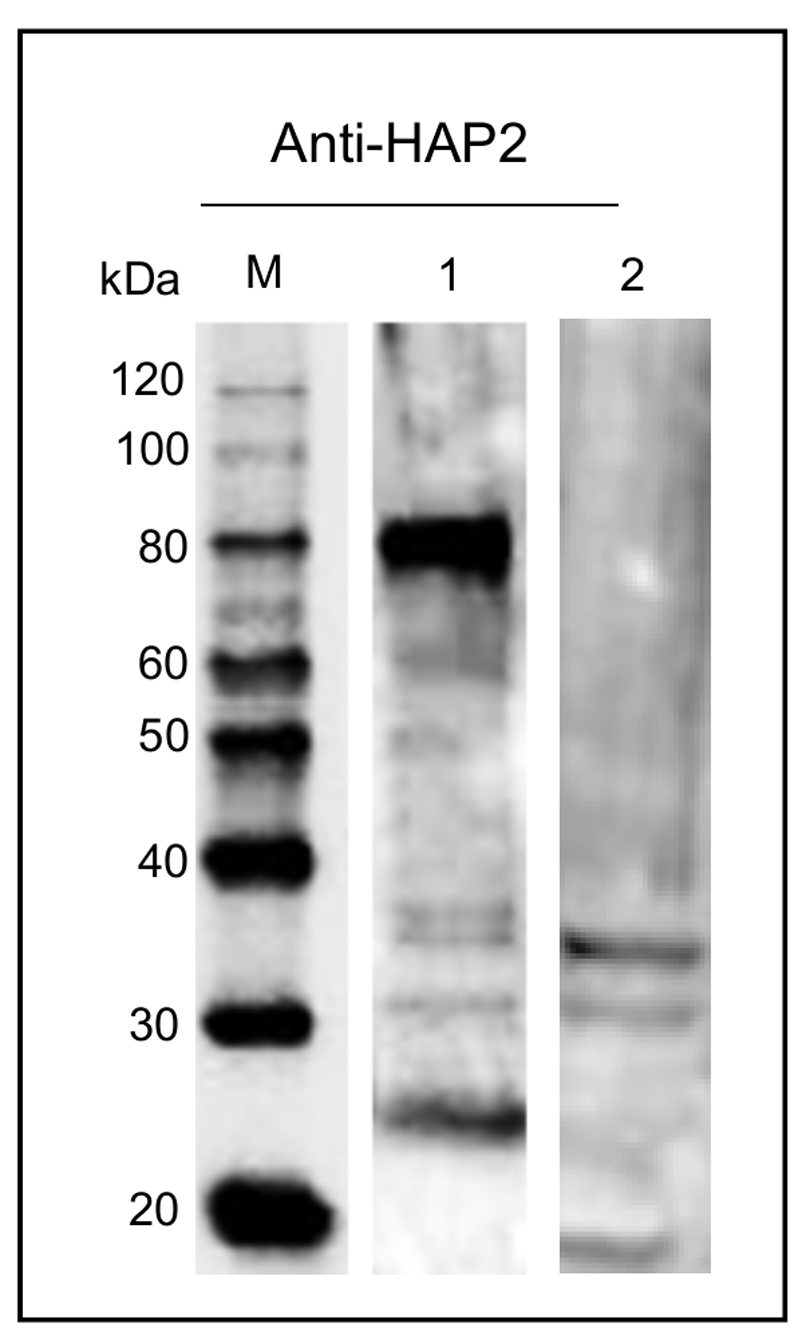

Supplement: S1 Fig — Lane 1: Bacterial lysate derived from arabinose induced recombinant bacteria; Lane 2: Bacterial lysate derived from non-arabinose induced recombinant bacteria. Size markers (M) in kDa are indicated at the left side. (TIF) [file pntd.0005965.s001.tif]

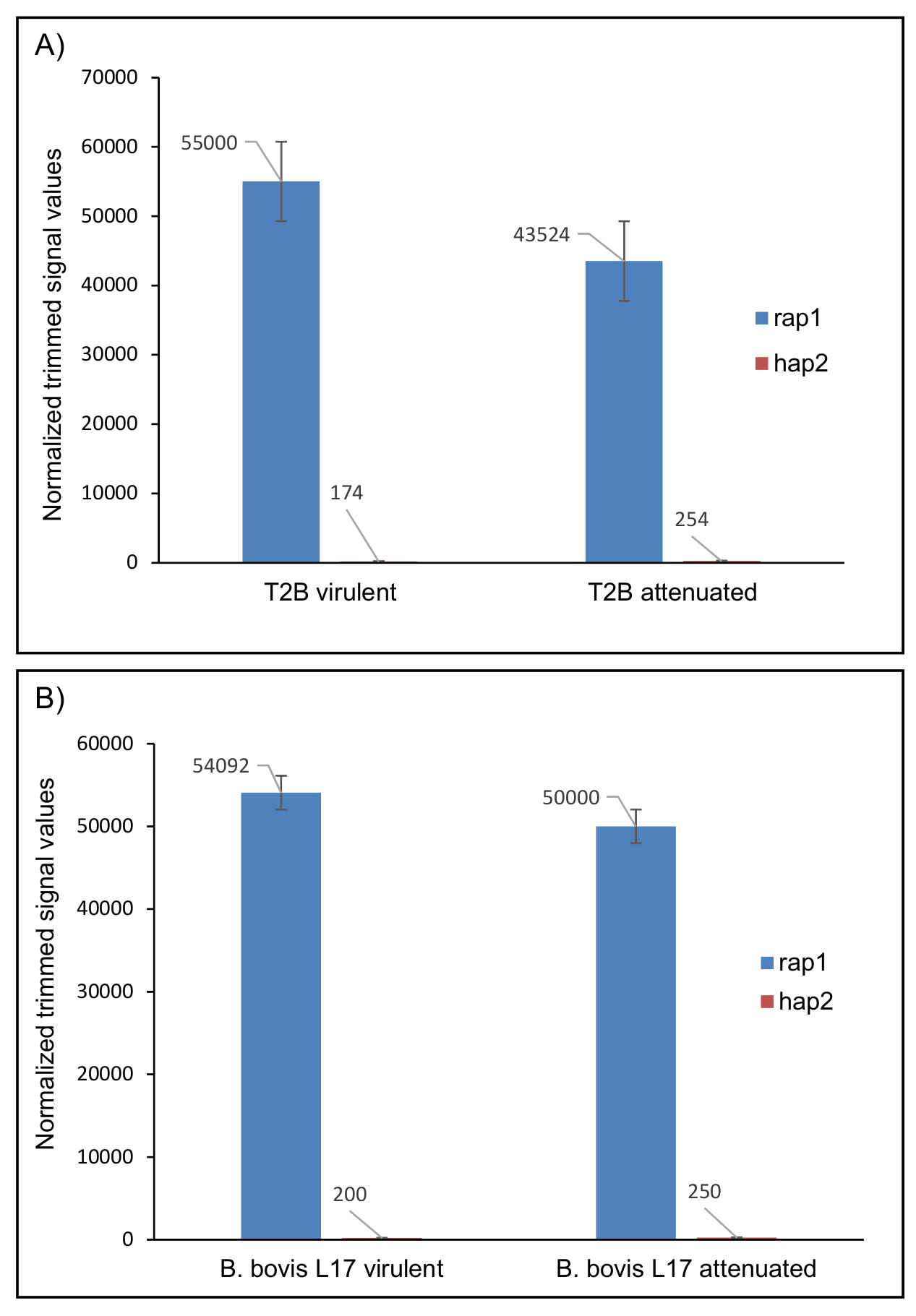

Supplement: S2 Fig — A. Microarray analysis of the virulent and attenuated parasites derived from B. bovis T2B strain. The Y axis indicates relative transcriptional levels. The X axis represents the name of the parasite strain. B. RNA seq analysis performed on the virulent and attenuated parasites derived from B. bovis L17 strain. The Y axis indicates relative transcriptional levels. The X axis represents the name of the parasite strain. (TIF) [file pntd.0005965.s002.tif]

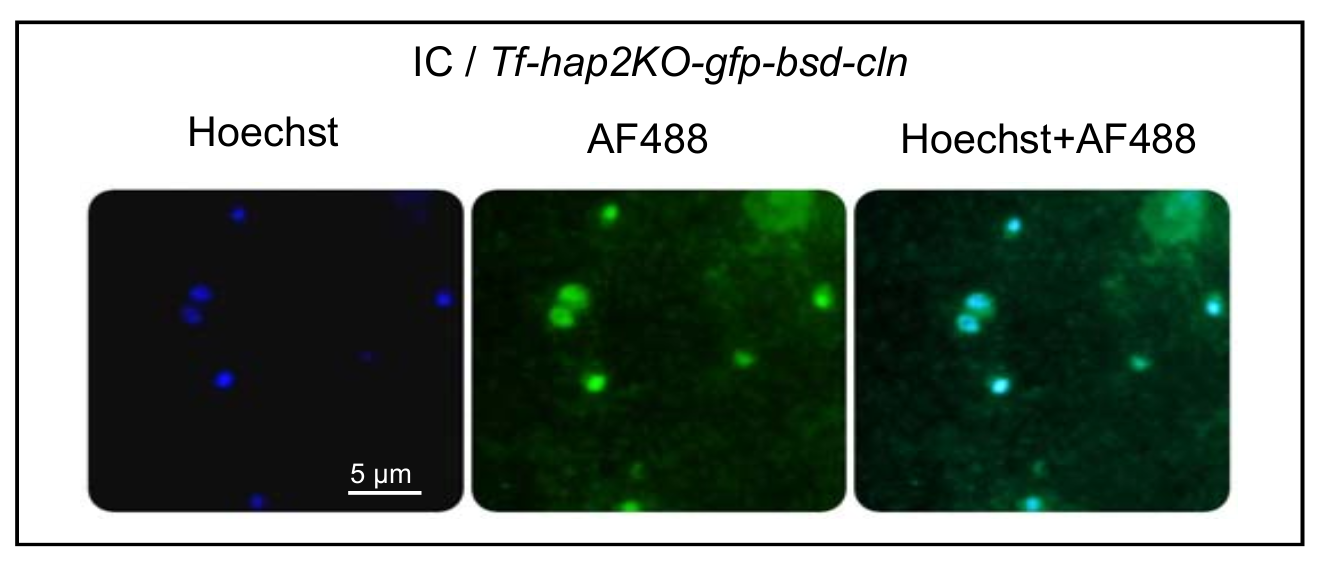

Supplement: S3 Fig — (TIF) [file pntd.0005965.s003.tif]

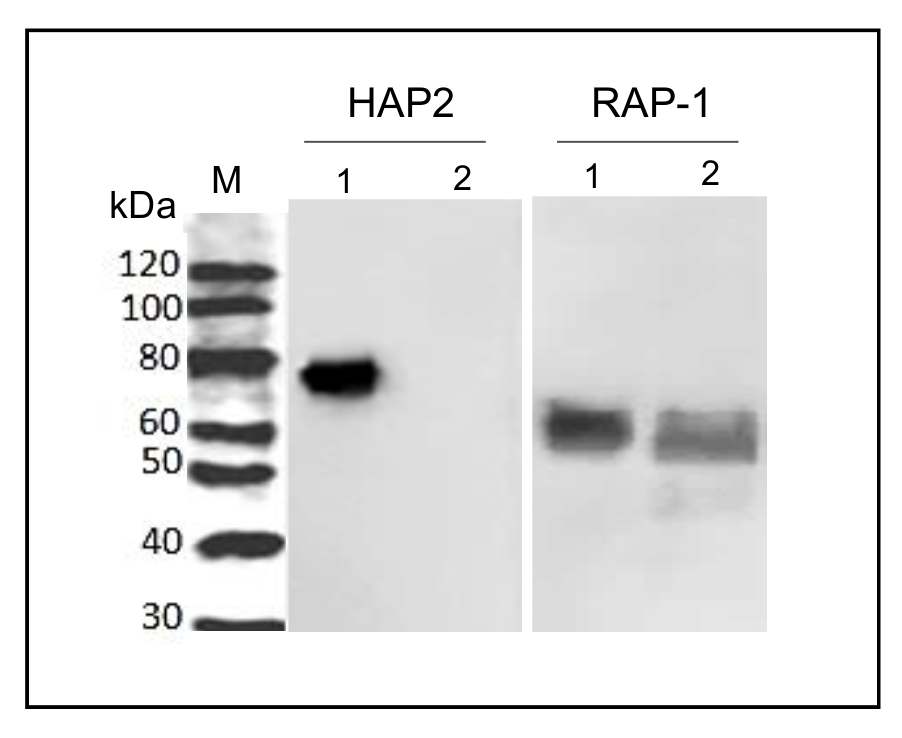

Supplement: S4 Fig — Monoclonal RAP-1 antibodies were used to detect B. bovis RAP-1 protein as a positive control. Size markers (M) in kDa are indicated at the left side. (TIF) [file pntd.0005965.s004.tif]
